# Personality traits of university students with smartphone addiction

**DOI:** 10.3389/fpsyt.2023.1083214

**Published:** 2023-02-08

**Authors:** Ali Kheradmand, Elham Sadat Amirlatifi, Zahra Rahbar

**Affiliations:** ^1^Department of Psychiatry, Taleghani Hospital Research Development Committee, Medical School, Shahid Beheshti Medical University, Tehran, Iran; ^2^Northamptonshire Healthcare Foundation Trust, Child and Adolescent Mental Health Service, Kettering, United Kingdom; ^3^School of Medicine, Shahid Beheshti University of Medical Sciences, Tehran, Iran

**Keywords:** smartphone addiction, personality traits, temperament, character, students

## Abstract

**Background:**

Nowadays smartphone use is increasing drastically. There is a higher prevalence of smartphone addiction in some specific personality traits.

**Objectives:**

The goal of this study is to evaluate the association of smartphone addiction with personality traits.

**Methods:**

This study is correlational research. Three hundred and eighty two students of Tehran universities were asked to answer the smartphone addiction scale (SAS) questionnaire and the Persian version of the Cloninger temperament and character inventory (TCI) questionnaire. After the smartphone addiction questionnaire assessment, individuals with smartphone addiction were identified and compared to the non-smartphone addicted group in terms of personality traits.

**Results:**

One hundred and ten individuals (28.8%) were prone to smartphone addiction. Mean scores of people with smartphone addiction were higher in novelty-seeking, harm avoidance, and self-transcendence than the non-addicts and were statistically significant. In persistence and self-directedness, the mean scores of the smartphone addiction group were lower than the non-addicts and were statistically significant. Individuals with smartphone addiction had higher reward dependence and lower cooperativeness however they were not statistically significant.

**Conclusions:**

high novelty seeking, harm avoidance, self-transcendence, low persistence, and self-directedness which indicate narcissistic personality disorder, could have a role in smartphone addiction.

## Introduction

In recent decades smartphone usage has increased dramatically worldwide. According to the PEW research center, about 94% of adults in developed countries use mobile phones, and 76% use smartphones (1). In today's world, smartphones have become multifunctional devices to make phone calls, surf the internet, use social media, etc. Moreover, their reasonable size and the fact that they are easy to use, have caused us to spend many hours on them in a day and numerous studies have described smartphone use dependency as addictive behaviors (2). Studies show there is a link between smartphone dependency and low self-esteem, impulsive behaviors, poor academic performance, alcohol abuse, excessive stress, and psychiatric disorders such as depression, anxiety, and PTSD (post-traumatic stress disorder) (3–5). Smartphone addictions could also cause blurry vision, neck pain, wrist pain, and impaired sleep quality (2, 6).

Recently the association between smartphone addiction and personality traits has been noticed by researchers. Some studies suggest that the prevalence of neuroticism and extraversion in people with smartphone addiction is significantly higher than in those who are not addicted to smartphones (7, 8). Another study suggests there is a negative correlation between conscientiousness, emotional stability, and problematic smartphone use, meaning the less conscientious, the less emotionally stable people are and the more mood swings they have, the more they are prone to problematic smartphone use. Also, it suggests people with a low desire to gain new experiences and new ideas are more likely to become problematic smartphone users. However, this study did not find any links between the two traits of neuroticism and extraversion and problematic smartphone use (9).

Another study showed people with smartphone addiction have more novelty seeking and harm avoidance among personality traits in which novelty seeking is associated with disorderliness and impulsivity and harm avoidance is associated with fear of uncertainty and shyness with strangers (6). Even In another study, high scores of narcissism were linked to smartphone dependency (10). Given the high prevalence of smartphone addiction and the role of personality traits in smartphone addiction, an assessment of the association between smartphone addiction and personality traits is necessary. There are a few studies in this field which they showed an association between some personality traits and smartphone addiction however they had different and various results and further evaluations are required. Our study intends to evaluate the association between smartphone addiction and personality traits in university students in Tehran.

## Materials and methods

This study was a correlational type of research conducted on 382 students of different faculties of universities in Tehran in 2019. Three hundred and eighty two students from different universities in Tehran were chosen by multi-stage cluster sampling. At first, we selected different universities from different regions of Tehran (South, North, East, and center), then we collected equal samples from each university, and then from each sample, equal samples of different faculties were collected. At last, we collected equal samples randomly from each faculty. After taking informed consent from the students, the following questionnaires were distributed among them: smartphone addiction scale questionnaire (SAS), temperament and character inventory (TCI), Young internet addiction test, and a questionnaire containing variables such as gender, age, internet use on a smartphone, use of game applications, use of social media, history of mental illnesses, drug abuse and some habitual behaviors. After evaluating the smartphone addiction scale questionnaire, individuals with smartphone addiction were identified and compared with non-smartphone addicts in terms of personality traits and other underlying variables.

### Assessment tools

#### Smartphone addiction scale (SAS)

This questionnaire contains 6 subscales which are daily life disturbance, positive anticipation, withdrawal, overuse, tolerance, and cyberspace-oriented relationship. It has 33 items overall and the total score calculated would be 48–288. The higher the score, the greater the scale of pathological and problematic smartphone use (11, 12). This questionnaire is validated and standardized for the Iranian population in a recent study. Based on its results best cut-off point was 106, meaning if an individual's SAS score is higher than 106, he is in danger of smartphone addiction. At this cut-off point, the questionnaire's sensitivity was 80% and the specificity was 86%. In this study by Kheradmand et al., the concurrent validity was assessed by a comparison between the Persian SAS and the Persian version of the Internet addiction test (IAT. The Persian SAS had a significant positive correlation with the Persian IAT with Pearson's correlation coefficient of 0.7 (*P* = 0.00), showing a good concurrent validity. Persian SAS also had good internal consistency with Cronbach's alpha of 0.93. Test-retest reliability of the Persian SAS was also high with an interclass correlation of 0.996. In a conclusion, the Persian SAS is a valid and reliable questionnaire to use in our study (13).

#### Temperament and character inventory (TCI)

The Persian version of TCI was used for this study. In 2005 the psychometric properties of TCI for the Iranian population were studied by Kaviani and Naseh. Cronbach's alpha fluctuated in the range of 0.55 for the persistence scale to 0.84 for the self-directedness scale (14).

The TCI which is designed by Cloninger evaluates an individual's personality in terms of temperament and character. The temperament part of this questionnaire includes 4 subscales that are novelty seeking, harm avoidance, reward dependence, and persistence. The character part includes 3 subscales of self-directedness, cooperativeness, and self-transcendence. Cloninger offered a model to evaluate the structure and evolution of personality which includes 2 dimensions of temperament and character.

Temperament with the 4 mentioned subscales is hereditary and presents itself early in life, hereditary and automatic reactions that are involved in associative learning and forming habits.

1. Novelty seeking is a temperament factor associated with excitability, impulsivity, emotional behaviors, a tendency to explore unfamiliar places and situations, and disorderliness. People with high novelty seeking are eager for changes, hate monotony, love exploration, and are curious.
2. Harm avoidance is a temperament factor associated with fear of uncertainty, shyness with strangers, fatigue, and being worried and pessimistic about the future.
3. Individuals with high reward dependence are sentimental, warm, dependent, kind-hearted, and sociable.
4. Persistence is the tendency to work hard and strive despite failure and exhaustion, be ambitious, and keep trying to achieve success.

Character dimension includes different individual traits which arise from knowing oneself and self-concept. They usually evolve in adulthood and are a reflection of one's goals and values that are obtained by knowing his/her identity independently, as part of society, and as part of the world.

1. Self-directedness is a character trait associated with responsibility, professionalism, self-sufficiency, and commitment to goals and aspirations.
2. Cooperativeness represents compassion, empathy, tolerance, patience, and social acceptance. People with high cooperativeness enjoy helping others.
3. Self-transcendence refers to the acceptance of spirituality. People with high scores in this field transcend beyond themselves and are patient and spiritual (15–17).

#### Young internet addiction test

This questionnaire was designed by Kimberly Young in 1998. It contains 20 items and assesses different aspects of internet overuse such as dependence, daily life, and social life disturbance. Its scoring system is by Likert scale. Each item is scored from 0 to 5 (the least extreme behavior to the most extreme behavior). A total score of 30 or less indicates normal internet use, a total score of 31–49 is mild addiction, 50-79 is moderate addiction and 80 and higher indicates severe internet addiction (18). However, in a study conducted by Alavi et al., a cut-off point of 46 was obtained and suggests that a total score higher than 46 indicates internet addiction. Alavi et al. also determined the content and convergent validity, internal consistency with Cronbach's alpha of 0.88, and test-retest reliability with a correlation coefficient of 0.82 of the Persian Young internet addiction test showing that the Persian version of the Young internet addiction test is a valid and reliable tool to assess internet addiction (19).

### Sample size calculation

The study population was the students of Tehran universities and sampling was done by multi-stage cluster method. Assuming a population proportion (*p*) of 50%, a confidence level of 95% and *Z*-score of 1.96, and a margin of error (*d*) of 5% the sample size was calculated about 382 people.

### Statistical analysis

Statistical data were gathered using SPSS-22 software. Frequency was reported descriptively. The *T*-test was used for quantitative variables and the chi-square test was used for qualitative variables.

### Ethical considerations

In this study, participants were first given sufficient explanations about the research. In case individuals were unwilling to participate they were excluded from the research. Written consent was obtained from all participants. The questionnaires were nameless and all data were confidential. This research has been registered in the ethics committee of the medical school of Shahid Beheshti University of medical sciences and the ethics code is 1395 309.

## Results

The study population was 382 university students from different faculties of Tehran University. 148 (38.7%) of the participants were male and 234 (61.3%) of them were female. 58.6% were 20–29 years old. The rest of the demographic features of the participants are mentioned in Table 1. 110 individuals (28.8%) were at risk of smartphone addiction based on the SAS questionnaire with a cut-off point of 106. The prevalence of smartphone addiction is higher in women (32.5%) than in men (23%) which is a statistically significant difference based on the chi-square test with a *P*-value of 0.04. The highest prevalence of smartphone addiction was in individuals under 20 years old and the lowest prevalence was in individuals over 40 years old which was statistically significant based on the chi-square test with a *P*-value of 0.01.

**Table 1:** Demographic features of the students of Tehran universities.

**Variables**		**Frequency (percentage)**
Gender	Male	148(38.7)
	Female	234(61.3)
Age	20>	26(6.8)
	29–20	224(58.6)
	39–30	76(19.9)
	40<	56(14.7)
Marital status	Single	270(70.7)
	Married	112(29.3)
Field of study	Medical science	216(56.5)
	Engineering	40(10.5)
	Basic science	18(4.7)
	Liberal science	108(28.3)
Level of education	Associate degree	24(6.3)
	Bachelor degree	88(23)
	Master degree	60(15.7)
	Doctoral degree/higher	210(55)

Simultaneous internet addiction, whether the phone is equipped with the internet or not, use of game applications, and use of social media were all compared in two groups of smartphone addiction and non-smartphone addiction in our participants, and the results are mentioned in Table 2.

**Table 2:** Comparison of simultaneous internet addiction, the phone being equipped with internet, using game applications, and using social media in two groups of smartphone addiction and non-addiction in Tehran's university students.

**Variables**		**Smartphone addicted group**	**Non-smartphone addicted group**	**Overall**
Internet addiction	Yes	64 (16.7%)	16 (4.1%)	80 (20.8%)
	No	46 (12%)	256 (67%)	302 (79%)
Is the phone equipped with the internet?	Yes	110 (28.7%)	260 (68%)	370 (96.7%)
	No	0 (0%)	12 (3.1%)	12 (3.1%)
Use of game applications	Yes	64 (16.7%)	130 (34%)	194 (50.7%)
	No	46 (12%)	142 (37.1%)	188 (49.1%)
Use of social media	Yes	110 (28.7%)	260 (68%)	370 (96.7%)
	No	0 (0%)	12 (3.1%)	12 (3.1%)

### Comparison of personality traits based on smartphone addiction

#### Novelty seeking

The mean scores of novelty seeking were higher in smartphone-addicted individuals than in non-addicts and this difference was statistically significant based on the independent *T*-test (*P* = 0.00).

#### Harm avoidance

The mean scores of harm avoidance in people with smartphone addiction were higher than in people who were not addicted and this difference was statistically significant based on the independent *T*-test (*P* = 0.001).

#### Reward dependence

Individuals with smartphone addiction had more reward dependence than people with no smartphone addiction but this difference was not statistically significant (*P* = 0.062).

#### Persistence

In persistence, the mean scores in the smartphone-addicted group were lower than in non-addicts and this difference was statistically significant (*P* = 0.032).

#### Cooperativeness

The mean scores of cooperativeness were lower in addicts than in non-addicts but this difference was not statistically significant (*P* = 0.185).

#### Self-directedness

In self-directedness, the average scores were lower in individuals with smartphone addiction than in individuals with no addiction and this difference was statistically significant based on the independent *T*-test (*P* = 0.000).

#### Self-transcendence

People with smartphone addiction had higher scores in self-transcendence than people who were not addicted and this difference was statistically significant (*P* = 0.024).

The results of the comparison of personality traits based on smartphone addiction are mentioned in Table 3.

**Table 3:** Comparison of personality traits based on smartphone addiction in Tehran's university students.

**Personality traits**	**Smartphone addiction**	**Frequency**	**Mean score**	**Standard deviation**	**Standard error**	***P*-value**
Novelty seeking	Addicts Non-addicts	110 272	49.6984 45.2637	10.48474 8.69659	0.99968 0.52731	0.00
Harm avoidance	Addicts Non-addicts	110 272	50.5164 46.2631	12.43211 11.37477	1.18536 0.68970	0.001
Reward dependence	Addicts Non-addicts	110 272	46.8298 45.3749	7.33804 6.69286	0.69965 0.40581	0.062
Persistence	Addicts Non-addicts	110 272	30.3291 31.2703	4.02113 3.80210	0.38340 0.23054	0.032
Cooperativeness	Addicts Non-addicts	110 272	67.1235 69.0628	13.51069 11.26334	1.28819 0.68294	0.185
Self-directedness	Addicts Non-addicts	110 272	54.3213 63.0436	12.64557 12.86941	1.20571 0.78032	0.000
Self-transcendence	Addicts Non-addicts	110 272	45.6753 43.2936	9.77210 9.06317	0.93173 0.54954	0.024

## Discussion

### The association of personality traits and smartphone addiction

The results of this study suggest that smartphone addiction is significantly related to high novelty seeking, high harm avoidance and low persistence of the temperament's dimensions, and low self-directedness of the character's dimensions.

The results of our study in terms of a positive correlation between high novelty seeking, high harm avoidance and smartphone addiction is in line with the results of Lane et al.'s study. They demonstrated that high scores in impulsivity and disorderliness of novelty seeking and high scores in fear of uncertainty and shyness with strangers of harm avoidance are significantly associated with smartphone addiction (6). Also, in a study by Kim et al., it was concluded that smartphone addiction is associated with impulsivity, low self-control, and high reward dependence (20). However in our study, high scores in reward dependence in individuals with smartphone addiction were not statistically significant.

Our study suggests that smartphones with different social media platforms create the opportunity of meeting online friends, share photos, viewing likes and comments of other users, and hence satisfy the need of seeking attention in people with low self-confidence and narcissistic backgrounds. One study suggested narcissistic vulnerability has positive correlations with harm avoidance and novelty seeking and negative correlations with reward dependence, cooperativeness, and self-directedness. Considering that our results in terms of personality patterns are in line with this pattern, it could be suggested that smartphone addiction is somehow associated with narcissism (21). This was consistent with the results of a study by Pearson et al. that concluded high scores in narcissism were associated with smartphone addiction (10).

Several studies have emphasized the association between neuroticism and smartphone addiction (7, 8, 22). People with high neuroticism experience negative emotions such as anxiety, anger, and frustration more than others. They don't respond appropriately to environmental stressors and become emotionally unstable and anxious (23). In our study people with smartphone addiction obtained higher scores in ham avoidance than people with no addiction which could indicate underlying anxiety disorder in people with smartphone addiction. The correlation between neuroticism and harm avoidance has already been shown by previous studies (24).

There could also be an association between smartphone addiction and ADHD (attention deficit hyperactivity disorder). One study found that high novelty seeking, high harm avoidance, and high self-transcendence as well as low self-directedness and low cooperativeness, are associated with ADHD (attention deficit hyperactivity disorder) patterns (25). Also, another study found a link between high novelty seeking, high harm avoidance, low persistence, and ADHD (26). We found the same pattern in our study as well, higher scores in novelty-seeking, harm avoidance, and self-transcendence, as well as lower scores in self-directedness and persistence, were obtained in the smartphone addiction group than in the non-addiction group which was all statistically significant, on the other hand, cooperativeness scores were lower in the smartphone addiction group than in the non-addiction group but this difference was not statistically significant. High harm avoidance and low self-directedness could also indicate a pattern of depression and mood disorders (27–29). According to these results, it could be indicated that there is an association between smartphone addiction and depression and ADHD which is also in line with several studies (5, 30).

In general, the results of this study suggest that people with smartphone addiction show high amounts of novelty seeking, harm avoidance, and self-transcendence as well as low amounts of persistence and self-directedness. These personality patterns could be associated with narcissism, ADHD, depression, and mood disorders. Knowing the personality pattern of someone prone to smartphone addiction would be a big help to prevent the addiction. Due to all the harms of smartphone addiction, it is possible to prevent smartphone addiction and daily-life disturbance by identifying these patterns and treating them as soon as possible.

## Limitations

Four questionnaires including demographic features questionnaire (22 multiple choice and essay type questions), Young internet addiction questionnaire (20 multiple choice questions), smartphone addiction scale or SAS questionnaire (33 multiple choice questions), and Cloninger temperament and character inventory or TCI questionnaire (125 true or false questions) were distributed among participants. Due to the high number of questions, many questionnaires were answered incompletely, therefore, had to be excluded from the study. This problem made the sampling process (382 individuals) long and difficult. Also, this high number of questions may have affected the participants' accuracy in answering them.

This study was conducted among students who usually do not have severe dysfunctions. This problem makes it difficult to extend the results of this study to the clinical setting. We suggest future studies focus on clinical patients and the results be compared with the findings of this study.

Due to the high sample size of this study and the high number of questionnaires, it was not possible to use clinical interviews and DSM criteria. It is suggested that future studies reduce the number of questions and use clinical interviews and clinical assessments to diagnose psychiatric illnesses and personality disorders. This helps increase diagnostic accuracy and facilitates extending the findings to the clinical setting.

## Data availability statement

The original contributions presented in the study are included in the article/supplementary material, further inquiries can be directed to the corresponding authors.

## Ethics statement

The studies involving human participants were reviewed and approved by the Ethics Committee of the Medical School of Shahid Beheshti University of Medical Sciences. The patients/participants provided their written informed consent to participate in this study.

## Author contributions

AK contributed to the conception and design of the study. AK and EA organized the database. EA performed the statistical analysis. ZR wrote the first draft of the manuscript. EA and ZR wrote sections of the manuscript. All authors contributed to manuscript revision, read, and approved the submitted version.
